# A Sex-Specific Evaluation of Dental Students’ Ability to Perform Subgingival Debridement: Randomized Trial

**DOI:** 10.2196/44989

**Published:** 2023-04-28

**Authors:** Ariadne Charis Frank, Linda Jennrich, Philipp Kanzow, Annette Wiegand, Christiane Krantz-Schäfers

**Affiliations:** 1 Department of Preventive Dentistry, Periodontology and Cariology University Medical Center Göttingen Göttingen Germany

**Keywords:** dental, dental education, dentist, education, gender, periodontics, preclinical education, root debridement, sex, student

## Abstract

**Background:**

A successful periodontitis treatment demands good manual skills. A correlation between biological sex and dental students’ manual dexterity is currently unknown.

**Objective:**

This study examines performance differences between male and female students within subgingival debridement.

**Methods:**

A total of 75 third-year dental students were divided by biological sex (male/female) and randomly assigned to one of two work methods (manual curettes n=38; power-driven instruments n=37). Students were trained on periodontitis models for 25 minutes daily over 10 days using the assigned manual or power-driven instrument. Practical training included subgingival debridement of all tooth types on phantom heads. Practical exams were performed after the training session (T1) and after 6 months (T2), and comprised subgingival debridement of four teeth within 20 minutes. The percentage of debrided root surface was assessed and statistically analyzed using a linear mixed-effects regression model (*P*<.05).

**Results:**

The analysis is based on 68 students (both groups n=34). The percentage of cleaned surfaces was not significantly different (*P*=.40) between male (mean 81.6%, SD 18.2%) and female (mean 76.3%, SD 21.1%) students, irrespective of the instrument used. The use of power-driven instruments (mean 81.3%, SD 20.5%) led to significantly better results than the use of manual curettes (mean 75.4%, SD 19.4%; *P*=.02), and the overall performance decreased over time (T1: mean 84.5%, SD 17.5%; T2: mean 72.3%, SD 20.8%; *P*<.001).

**Conclusions:**

Female and male students performed equally well in subgingival debridement. Therefore, sex-differentiated teaching methods are not necessary.

## Introduction

Medical and dental professionals are required to perform a wide range of manual tasks as part of their clinical practice. It is essential for students to develop good manual dexterity skills through (virtual) training, as the dental education determines the quality of treatment in the dental practice [1]. The practical training of manual skills is challenging, not only for the students but also for teaching physicians [2]. Hence, training practical skills is a core part of dental education, and the examination and improvement of teaching techniques are vital for the enhancement of further teaching methods and substance.

The achievement of practical skills is an integral part of the dental undergraduate curriculum. Substantial research has been carried out to identify factors that might affect motor learning and the achievement of manual dexterity. Several previous studies addressed sex and age as potential factors affecting motor learning and motor performance [3-5]. However, the internal processes of motor learning depend not only on functional characteristics or anthropometrics that might differ between sexes or ages but also on neurological differences depending on sex or changing with age. With regard to fine motor skills, conflicting results regarding potential sex-related differences have been found. Some studies found a male advantage in speed but not in accuracy, while the performance of more complex tasks (like mirror drawing) or hand stability was better in women compared to men [6-8]. However, in medical or dental education, potential sex-related differences in achieving certain manual skills were rarely investigated so far. Kolozsvari et al [9] found no sex-specific performance differences after examining laparoscopic skill among medical students. Another study evaluating the surgical skills of medical students reported better performance of the female students compared to their male counterparts [10].

For the treatment of periodontitis, the reduction and disintegration of microbial biofilm on tooth surfaces and within periodontal pockets are key for minimizing the infectious condition [11]. The procedure, called deep scaling or subgingival debridement, is usually carried out using (manual) curettes or power-driven instruments. Both methods demand good manual skills and cognitive abilities, and studies have shown both methods to be equally efficient [12,13]. Dental students learn and practice treatment procedures using dental simulators or phantom heads before proceeding to treat patients. This training includes clinical tasks, such as root debridement, as a part of periodontology treatments.

Studies have indicated that the use of hand instruments by untrained practitioners may cause inadequate debridement and unwanted roughness of the root surface [13,14]. For this reason, repetitive practicing on models is essential for students’ training in periodontics. Among medical students, work experience has been shown to correlate with enhanced surgical skills [15]. However, the question of whether or not biological sex influences the practical skills of treating physicians has been debated for many years [16]. Studies examining cognitive patterns with regard to biological sex have been conducted and various findings reported. To the best of our knowledge, within the dental field, there have been no studies investigating a correlation between biological sex and students’ manual skills. Given the dearth of knowledge about sex-related differences (if any) in manual skills among dental students, this study aims to investigate performance differences between male and female students in subgingival debridement.

The null hypotheses were that biological sex and the applied work method do not result in performance differences within subgingival debridement.

## Methods

### Ethics Approval

The study was approved by the local ethics committee of the University Medical Center Göttingen (approval number: 21/10/18), and all students gave written informed consent before being enrolled in the study.

### Trial Design

This prospective intervention study is a randomized trial, evaluating the performance of dental students with regard to their sex and the instrument used to carry out a specific task. The study was conducted in accordance with the CONSORT (Consolidated Standards of Reporting Trials) guidelines [17]. The CONSORT checklist is available in Multimedia Appendix 1.

### Participants and Preparations

The study participants were third-year dental students attending the preclinical phantom course in Operative Dentistry in the summer term of 2019 and winter term of 2020/2021 at the University Medical Center Göttingen. The ongoing global COVID-19 pandemic resulted in restricted course regulations that, in turn, precluded the winter term 2019/2020 and summer term 2020 classes from being included in the study. Students were inexperienced with regard to root debridement, as periodontology was not part of the previous curriculum. The students were inquired about other training or experience they might have had (eg, training as a dental assistant or dental technician). This information was taken into consideration in the statistical analysis.

Lessons in the theoretical foundations and procedures of periodontics were given as usual.

### Interventions

The group of study participants was divided by biological sex (male/female). None of the participants defined themselves as nonbinary or were intersex. Following this, they were randomly assigned one of two work methods: the manual use of Gracey-curettes (HuFriedy, United States; No. 5/6, 7/8, 11/12, 13/14) or the use of power-driven instruments (KaVo, Germany; Sonicflex 2003 L, No. 61 and 62). The manual instruments were either new or appropriately sharpened before the initial use and both exams by trained staff. The study participants were instructed in the theoretical and practical use of the relevant instruments according to their assigned work method. A live video demonstration was performed by a senior clinician. On day 1, the participants practiced using their instruments under the supervision of trained staff for 60 minutes. Over the next 10 days of the course, practice time was limited to 25 minutes per day. The students worked on periodontal models (Frasaco, Germany; A-PZ), which imitate a set of teeth with calculus and concrements. These models accurately replicate the anatomical features of gums and teeth, allowing dental students to practice periodontal treatments, such as scaling and root debridement. The hard deposits on the root surface were replicated using colored nail polish (2 layers). The simulation models were mounted to patient dummies with face masks, ensuring a realistic operating principle.

At the end of the study, all participants learned the other root debridement method.

### Outcomes

**Figure 1:**
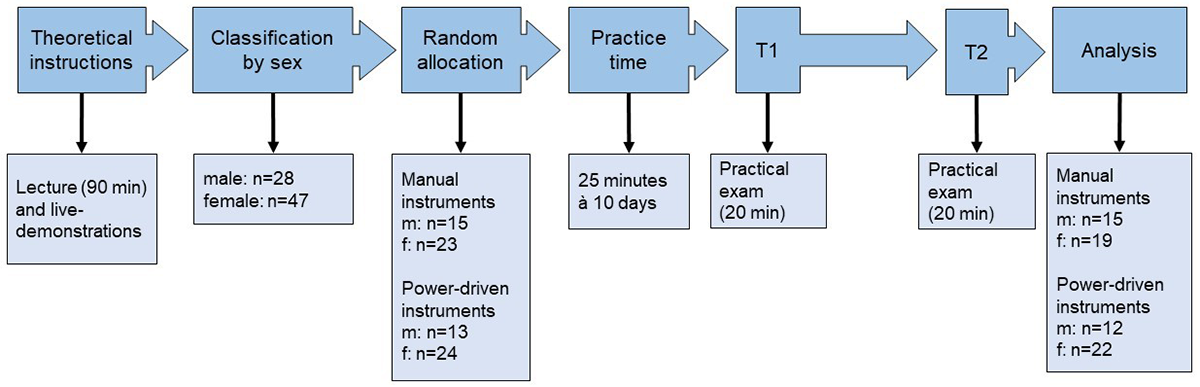
Timeline. f: female; m: male.

The skills exhibited by the students were evaluated twice over the course of the study (Figure 1). The students completed a practical formative exam directly after having practiced the debridement procedure for 10 days (T1) and were evaluated again 6 months later (T2), at which time they had to scale the roots of the following four teeth: 11, 26, 37, and 44. The teeth were thoroughly cleansed and coated with black and matt nail polish (Essence, Germany, Shine last&go; Trend it up, Germany, Ultra matte top coat). This enabled a percentual evaluation of the removed varnish as the primary outcome. To detect overly excessive treatment of the root surface and unwanted damage caused, an analysis by weight was done as a secondary outcome. The teeth were weighed before and after being coated with varnish and, at the end of the scaling procedure, using a microscale (Sartorius, Germany; MC1, Analytic AC 210 P).

**Figure 2:**
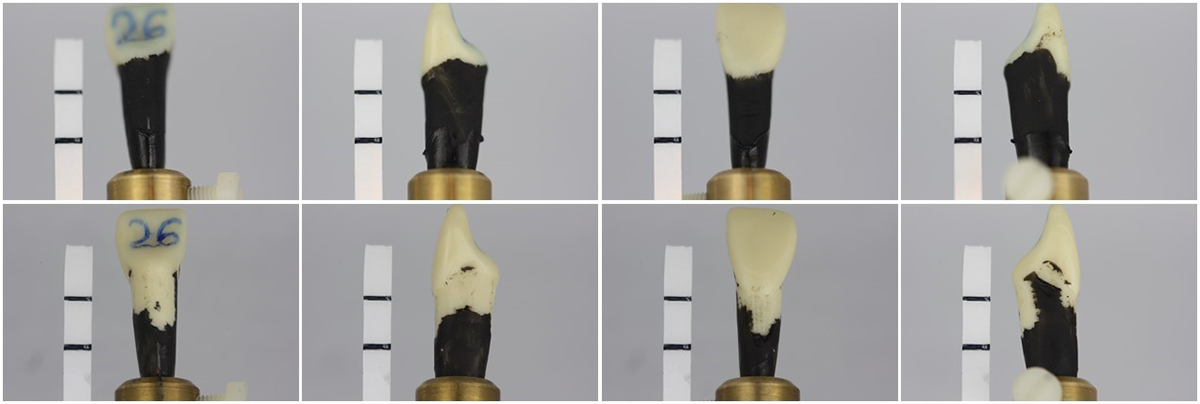
Varnished plastic tooth before and after root debridement; the white bar marks the ledge of the alveolar bone and the cement-enamel junction.

After applying the varnish, they were screwed back into the periodontal models. For the exam, the students were given 20 minutes to remove the nail polish by scaling the root surfaces, either manually or with power-driven instruments, according to the group to which they had been randomly assigned, as described above. The simulation models were collected, and the teeth were photographed from all sides (oral, mesial, vestibular, distal) directly upon completion of the exam (Figure 2).

The results were recorded by taking photographs of the teeth. The root surface to be examined was defined on reference teeth by drawing on the ledge of the alveolar bone and the cement-enamel junction using a mechanical pencil. These reference teeth were used and photographed for every exam. The photographs were taken using a digital camera (Canon, Japan; Canon DS126181) set to “M” (exposure time: 1/100 s; f/2.8). The teeth were secured in a fixture that allowed them to be turned to an angle of exactly 90°, enabling photographs of all root surfaces (mesial, distal, buccal, lingual). The surrounding sides and backdrop were white and were lit using two softboxes (ETiME, Germany; 5500 K Daylight). The relative amount of residual varnish was calculated using the program ImageJ (National Institutes of Health).

### Randomization

First, the study group was divided into two groups based on their sex (male and female). After that, the students were randomized to one of two study arms by blindly drawing a work method: manual curettes or power-driven instruments.

### Statistical Analysis

Statistical analyses were performed using the software R (version 4.1.2; R Foundation for Statistical Computing) [18] and the packages “lme4” (version 1.1-28) and “afex” (version 1.0-1).

The effect of the student’s sex on the removed varnish (%; primary outcome) and the evaluation of an overly excessive treatment by weight (secondary outcome) were analyzed using a linear mixed-effect regression model. Sex (female or male), instruments (manual or power-driven), time, previous training (none, uncompleted dental assistant training, completed dental assistant training, dental technician training, or course repeater), tooth (11, 26, 37, or 44), tooth side (distal, mesial, oral, or vestibular), and the interaction between sex and time were entered as fixed effects. Repeated measures (ie, the different time points T1 and T2) were considered by modeling random intercepts and random slopes per participant.

The level of significance was set to α=.05.

## Results

**Figure 3:**
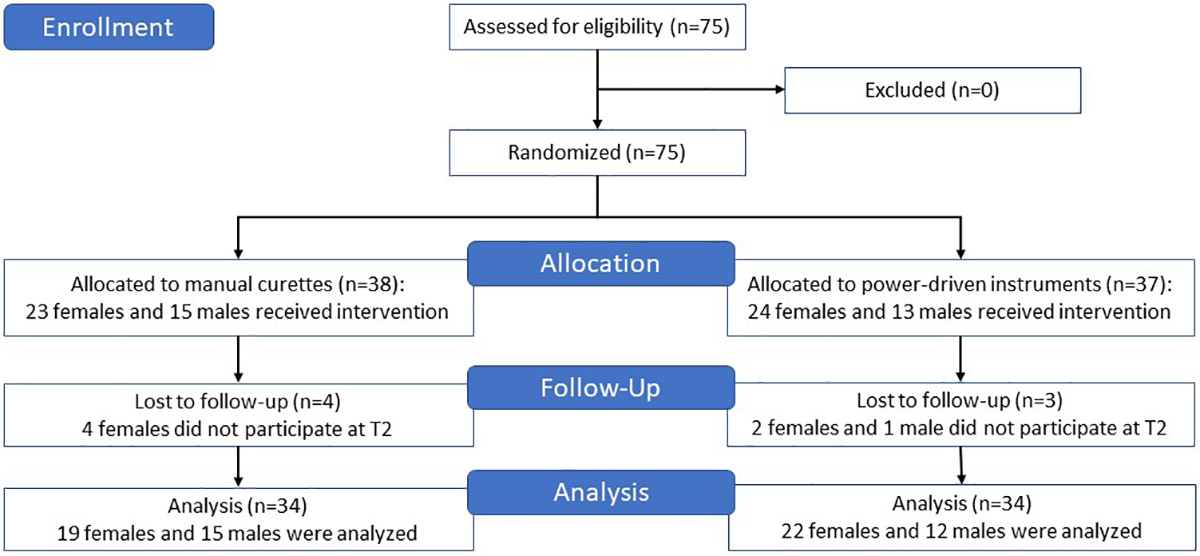
CONSORT (Consolidated Standards of Reporting Trials) study flowchart.

Overall, 75 students participated in the study (Figure 3). A total of 68 participants (41 women, 27 men) were included, after sorting out missing values (students who completed only one of the two practical examinations due to illness or other personal reasons). Considering the small number of dental students each year, this was an acceptable number of participants and resulted in significant outcomes. Altogether 19 students had prior experience (eg, dental assistant, dental technician, course repeater).

Male participants removed slightly, but not statistically significant, more varnish from the teeth than female students, irrespective of the instrument used. The use of power-driven instruments led to significantly better results than manual curettes. Overall, performance decreased significantly at T2. Furthermore, the vestibular and oral surfaces of the roots were cleaned significantly more thoroughly than the distal surface (*P*<.001). No significant differences between those with and those without prior experience were observed (Table 1). Additionally, the interaction between sex and time was not significant (*P*=.08).

As described previously, the teeth were weighed at three time points to detect possible overinstrumentation. Overall, the measured weight differences were small and, therefore, were possibly below the detection level. Prior to the study, a subsample of unworked teeth was repeatedly weighed (n=12). Thereupon an average SD of 0.00032 was calculated. As the examined teeth were weighed three times, the measurement error can be expected to amount to 0.00192 g. The mean weight differences were below this value; conclusively, the secondary outcome was no longer taken into account and no statistical analysis was performed.

Based on the results of the primary outcome, the first null hypothesis must be rejected.

**Table 1:** Reduction of simulated plaque.

Parameter and level		Removed varnish (%), mean (SD)	Effect estimate (%), 95% CI	*P* value
**Sex**				
	Female (reference group)	76.3 (21.1)	N/A^a^	N/A
	Male	81.6 (18.2)	2.628 (–3.20 to 8.51)	.40
**Instrument**				
	Manual (reference group)	75.4 (19.4)	N/A	N/A
	Power-driven	81.3 (20.5)	5.983 (1.14 to 10.82)	.02
**Time point**				
	T1 (reference group)	84.5 (17.5)	N/A	N/A
	T2	72.3 (20.8)	–13.881 (–16.78 to –10.98)	<.001
**Prior experience**				
	None (reference group)	79.0 (20.3)	N/A	N/A
	Dental assistant (uncompleted training)	70.7 (17.5)	–3.571 (–15.42 to 8.28)	.57
	Dental assistant (completed training)	72.1 (20.3)	–5.378 (–13.39 to 2.63)	.21
	Dental technician (completed training)	83.7 (17.7)	1.849 (–6.16 to 9.85)	.66
	Course repeater	78.1 (20.2)	–3.953 (–18.16 to 10.25)	.60
**Tooth**				
	11 (reference group)	78.7 (18.9)	N/A	N/A
	26	68.4 (22.9)	–10.224 (–11.78 to –8.67)	<.001
	37	86.0 (18.0)	7.350 (5.79 to 8.91)	<.001
	44	80.4 (16.2)	1.729 (0.17 to 3.29)	.03
**Tooth side**				
	Distal (reference group)	73.2 (22.2)	N/A	N/A
	Mesial	73.2 (20.7)	–0.002 (–1.56 to 1.56)	>.99
	Oral	80.1 (19.5)	6.889 (5.33 to 8.45)	<.001
	Vestibular	87.0 (14.1)	13.767 (12.21 to 15.32)	<.001

^a^N/A: not applicable.

## Discussion

### Principal Findings

This study investigated whether there are sex-specific performance differences in subgingival scaling procedures using manual as well as power-driven instruments. The results demonstrate that sex does not appear to be a significant factor in the performance of dental students regarding root debridement. The use of power-driven instruments led to significantly better outcomes irrespective of sex. Furthermore, systematic training is essential for obtaining proficiency in this matter, regardless of the used instruments.

### Comparison to Prior Work

Dorfberger et al [3] found men to benefit more from practice sessions than women and, furthermore, described men to have an advantage in procedural memory consolidation. Therefore, investigations at exclusively one time point may be prejudiced due to prior experience and training. Thus, for this study, performances were evaluated at different points of time. Results showed no significant differences regarding sex-specific performance. This applies to both investigated time points (T1 and T2). This finding resembles the results presented by Kolozsvari et al [9] who examined fundamental laparoscopic skill among medical students—a procedure that also demands a high degree of manual dexterity. Their results showed no sex-specific performance differences.

As many researchers have found women to be more precise and exact in their manual work than men, one could presume that hand size might be a factor to explain these observations [19]. A smaller hand size may facilitate manually working on a small scale and, in turn, result in better fine-motor performances among women compared to men. Peters and Campagnaro [20] conducted a study comparing the manual dexterity of women and men irrespective of their hand size by doing the O’Connor tweezer dexterity task. Study participants of both groups completed the tasks without significant differences in outcomes. In this study, female and male study participants used curettes of the same size and brand to carry out the given task. This, as in the study cited above, eliminated the hand size factor weighing into the results. Rohr [7] has shown that male subjects are faster at finger tapping, presenting a higher movement speed. The actual working speed was not investigated in this study, and thus, no direct comparisons can be drawn. However, all participants were given the same limited practice time and 20 minutes to perform the root debridement on four teeth. None of the students were willing to turn in their work before time. Further studies perhaps could investigate whether or not there is a sex-specific performance difference with regard to the work time.

Furthermore, in this study, power-driven instruments led to significantly better results than manual curettes. In a previous study, Graetz et al [21] observed that practitioners handling power-driven instruments work more ergonomically than those using hand instruments, irrespective of the operator’s level of experience. In addition to that, the use of hand instruments was described as more tiresome and demanding. On the other hand, however, other researchers found no significant differences in root debridement with regard to the instruments used [12]. Power-driven systems and manual Gracey curettes have been described as similarly easy to learn [22]. However, subgingival debridement usually is completed faster with powered instruments, and many clinicians prefer to use these [12,23]. Despite various researchers having made different observations on this matter, a common sentiment is that experience and training have a substantial effect on a practitioner’s performance [22,24,25]. In this study, most participants were equally unexperienced. Those who had stated that they had some sort of training in the dental field, however, did not perform significantly better. Most of those with prior experience had worked as either dental assistants or technicians. The study results show that this does not necessarily guarantee proficiency in periodontal treatments, despite the familiarity with procedures and tools. Root debridement requires a specific set of knowledge, skills, and practice that may not be part of their usual responsibilities. While they may have some exposure to periodontal procedures, their training and experience may not have focused on the detailed techniques for effective root debridement.

The drop in performance at T2 may be due to the disruption of practice time, as the students moved on with their course curriculum, which did not include further periodontics training. Therefore, the results from T2 display the participant’s performance with no practice time immediately before the evaluation. This also shows that constant training is crucial for satisfactory and optimal results. Untrained operators perform more poorly, irrespective of the used instrument [13], stressing the necessity of preliminary systematic training. Furthermore, inexperienced operators have been described to be more likely to cause damage to the root surface when using hand instruments [26]. This, however, could not be confirmed. In terms of overexcessive root debridement, this study did not display differences with regard to the used instruments. As the results from the weight analysis were lower than the scale’s detection limit, one can presume a minimal substance loss, if any. Graetz et al [24] found that receiving systematic training for chosen instruments may improve treatment results regardless of experience level. The participants of this study had received thorough instructions and had practiced root debridement while supervised over the course of 10 days. This may have had a positive effect on their initial performance.

Root debridement in niche and furcation areas is more difficult than on smooth surfaces. Yet, contrary to our expectations, the study participants displayed the best results for the tooth 37. Nonetheless, as done in other studies [26,27], one must take into consideration that the root surfaces were analyzed in a 2D array. Bearing in mind that the removal of the varnish is underestimated in furcation areas, this might explain the outcomes to a degree. Rühling et al [13] have observed that power-driven systems work less effectively on root surfaces with complex anatomy.

### Practical Implications

In comparison to power-driven instruments, the use of hand instruments enables the practitioner to have direct tactile control. For these reasons, weighing the pros and cons of the two devices, it seems reasonable to instruct students in the handling of both [12]. As previously mentioned, the participants of this study were taught the other root debridement technique subsequent to the examinations.

### Strengths and Limitations

Strengths of this study enhancing the reliability and validity of the findings include a relatively balanced fraction of male and female participants, ensuring the outcomes are representative of both sexes. The use of anatomical models of the same kind throughout the examination promotes comparability and reduces potential confounders that may affect the results. Furthermore, all students worked under the same circumstances (ie, models, instruments, time). This reduces the potential for extrinsic influences possibly affecting the outcomes.

However, there are also limitations present. First, due to restrictions caused by the COVID-19 pandemic, two semesters had to be precluded. For the remaining semesters during the COVID-19 pandemic, the theoretical part of the course was partially taught remotely; however, despite the COVID-19 pandemic, the practical course part was fully carried out and students were taught in cohorts [28]. Therefore, all those included in this study had completed the full practical curriculum of their studies. Consequently, one can assume that the pandemic did not have a considerable impact on the examined study group. Second, for the assessment and comparison to be as precise as possible, working on living patients was not applicable. Instead, periodontitis models were used enabling an accurate assessment of biofilm removal, and study participants worked on patient-like dummies. These models are commonly used for training purposes [29] and educational research [26,27]. As a precise assessment and, hence, comparison of subgingival biofilm removal is not possible in living patients, the use of phantom heads and periodontitis models seemed to be a suitable compromise, enabling a very accurate assessment of biofilm removal and an acceptable simulation of clinical conditions. The hard deposits on tooth and root surfaces were replicated using nail polish, which is a frequently used substance for similar examinations. Although varnish is not comparable to actual biofilm, it provides decent adherence and good visual feedback [30]. Similar products have been used in other studies and have displayed admissible results [14,31].

### Future Directions

Finally, to sum up, it can be said that as teaching methods are constantly being revised to enable an optimal education, knowledge of group-specific strengths and weaknesses may facilitate an adaption of teaching routines. The study results, however, indicate no appreciable performance differences between male and female dental students. There is no evidence for the necessity for sex-differentiated teaching methods in subgingival debridement.

### Conclusion

In conclusion, this study indicates that within root debridement, female and male dental students appear to perform equally well. Thus, it may be concluded that sex-differentiated teaching methods are not necessary. Nonetheless, systematic training is obligatory to adequately learn root debridement, irrespective of the instruments used.

## Appendix

Multimedia Appendix 1 CONSORT (Consolidated Standards of Reporting Trials) checklist.

## Abbreviations

CONSORT: Consolidated Standards of Reporting Trials

## Data Availability

The data sets generated or analyzed during this study are available from the corresponding author upon reasonable request.
